# Catechol 1,2-Dioxygenase is an Analogue of Homogentisate 1,2-Dioxygenase in *Pseudomonas chlororaphis* Strain UFB2

**DOI:** 10.3390/ijms20010061

**Published:** 2018-12-24

**Authors:** Boitumelo Setlhare, Ajit Kumar, Mduduzi P. Mokoena, Ademola O. Olaniran

**Affiliations:** Discipline of Microbiology, School of Life Sciences, College of Agriculture, Engineering and Science, University of KwaZulu-Natal (Westville Campus), Private Bag X54001, Durban 4000, South Africa; boitumellow5@gmail.com (B.S.); ajitkanwal@yahoo.com (A.K.); Mokoenap@ukzn.ac.za (M.P.M.)

**Keywords:** catechol 1,2-dioxygenase, homogentisate 1,2-dioxygenase, *Pseudomonas chlororaphis*, *Pseudomonas chlororaphis* strain UFB2

## Abstract

Catechol dioxygenases in microorganisms cleave catechol into *cis*-*cis*-muconic acid or 2-hydroxymuconic semialdehyde via the *ortho*- or *meta*-pathways, respectively. The aim of this study was to purify, characterize, and predict the template-based three-dimensional structure of catechol 1,2-dioxygenase (C12O) from indigenous *Pseudomonas chlororaphis* strain UFB2 (*Pc*UFB2). Preliminary studies showed that *Pc*UFB2 could degrade 40 ppm of 2,4-dichlorophenol (2,4-DCP). The crude cell extract showed 10.34 U/mL of C12O activity with a specific activity of 2.23 U/mg of protein. A 35 kDa protein was purified to 1.5-fold with total yield of 13.02% by applying anion exchange and gel filtration chromatography. The enzyme was optimally active at pH 7.5 and a temperature of 30 °C. The Lineweaver–Burk plot showed the *v*_max_ and *K*_m_ values of 16.67 µM/min and 35.76 µM, respectively. ES-MS spectra of tryptic digested SDS-PAGE band and bioinformatics studies revealed that C12O shared 81% homology with homogentisate 1,2-dioxygenase reported in other *Pseudomonas chlororaphis* strains. The characterization and optimization of C12O activity can assist in understanding the 2,4-DCP metabolic pathway in *Pc*UFB2 and its possible application in bioremediation strategies.

## 1. Introduction

The widespread distribution of aromatic compounds in the environment has led to an increase in pollution, which affects the health quality of living organisms [1]. Microorganisms have developed mechanisms to degrade these compounds with the aid of enzymes [2,3]. During the aerobic biodegradation of aromatic compounds, phenol, and it derivatives (e.g., 2,4-dichlorophenoxacetic acid (2,4-D) and 2,4-dichlorophenol (2,4-DCP)), catechols are formed as the central intermediates by the introduction of hydroxyl groups facilitated at *ortho*- or *meta*-positions [4,5,6]. The catechol is then oxidized via an *ortho*-cleavage pathway by catechol 1,2-dioxygenase (C12O), or via a *meta*-pathway to 2-hydroxymuconic semialdehyde by catechol 2,3-dioxygenase (C23O) to open the ring. The final intermediates of both pathways then enter the tricarboxylic acid cycle [7,8,9]. 

Catechol 1,2-dioxygenases have the potential to be used in the process of remediating wastewater contaminated with phenol, benzoate, fluorocatechol, bromocatechol, chlorocatechol, methylcatechol, herbicides (diuron), polychlorinated biphenyls, and chloroethanes [10,11]. The enzyme incorporates an oxygen atom into the catechol, resulting in the formation of *cis-cis*-muconic acid [12,13]. C12O is mostly reported in Gram-negative bacteria, but much less information is available about these enzymes in Gram-positive bacteria [10,12]. C12O contains Iron(III) oxide as a prosthetic group, and it is part of the enzymes that cleave catechol via the *ortho*-cleavage, resulting in the formation of *cis-cis-*muconic acid [14]. 

C12O was first isolated and purified in *Pseudomonas* spp. found to be dependent on Fe^2+^ and Fe^3+^ ions with high substrate specificity, showing molecular weight ranges from 22 to 35 kDa [13,15]. *Pseudomonas aeruginosa* TKU002 capable of mineralizing benzoic acid was reported to produce a low molecular weight C12O showing the highest activity against pyrogallol, which is an unusual characteristic [13,15]. *Trichosporon* sp. is reported to produce a high molecular weight C12O (100 kDa) that is stable at pH 8 but optimally active at pH 6.2 [13]. A non-heme ferric dioxygenase catalyzing the intradiol cleavage of all the examined catechol derivatives, 3,5-dichlorocatechol, was reported in *Pseudomonas cepacia* CSV90, grown with 2,4-D as the sole carbon source [16].

Enrichment studies on indigenous 2,4-DCP-degrading isolates from contaminated sites in Durban, South Africa showed an enormous potential to utilize 2,4-DCP as the sole carbon and energy source (unpublished data). The isolated culture was identified as *Pseudomonas chlororaphis* strain UFB2 (*Pc*UFB2) and found to exhibit phenol hydroxylase, catechol 1,2-dioxygenase, muconate isomerase, *cis*-dienelactone hydrolase, and *trans-*dienelactone hydrolase activities (unpublished data). 

Catechol, the intermediate in phenolic compound degradation pathways in bacteria, is also a derivative of benzene and a phenolic compound in many industrial applications, including as a photographic developer, lubricating oil, polymerization inhibitor, and in pharmaceuticals [17,18]. Catechol has a strong aroma and is a toxic and persistent water pollutant in the environment [17]. Thus, the aim of this study was to purify and characterize C12O in *Pc*UFB2 to understand the catalytic mechanism of the enzyme and predict its three-dimensional structure, for possible application of the enzyme in the removal of catechol from contaminated sites. 

## 2. Results

### 2.1. Production and Purification of C12O

**Figure 1:**
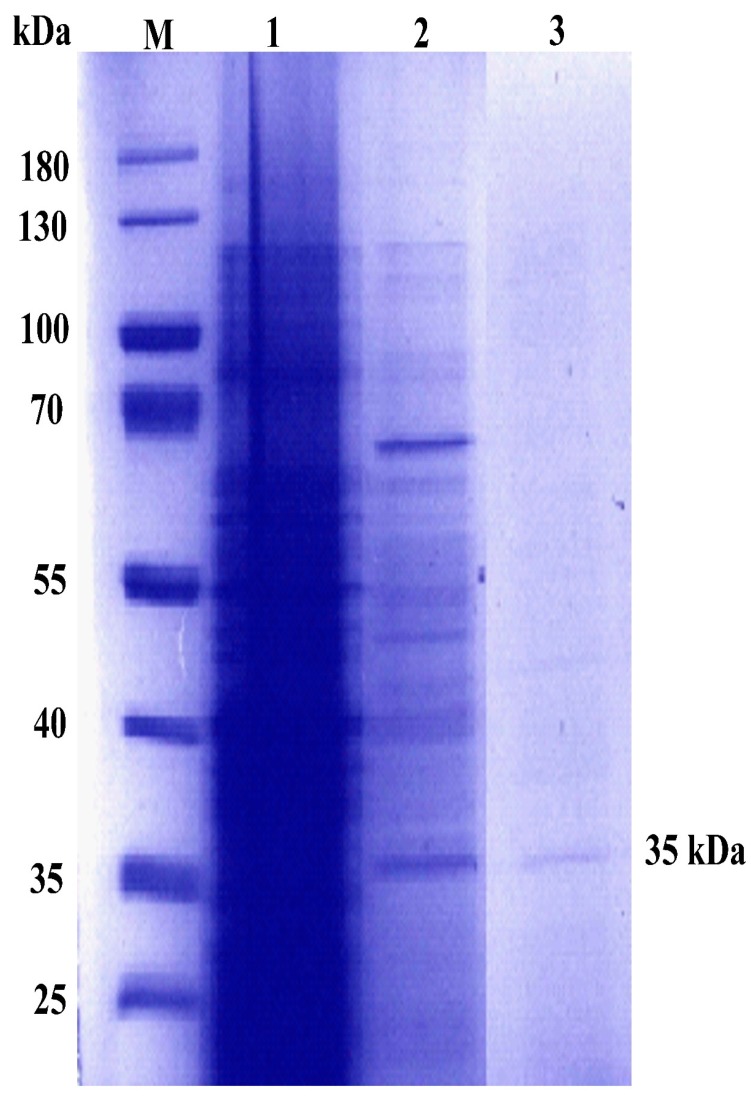
The 12% SDS-PAGE for crude and purified C12O from *Pc*UFB2. Lane M = protein Marker, Lane 1 = crude cell extract; Lane 2 = anion exchange fractions; Lane 3 = purified C12O from gel filtration fractions showing a band at 35 kDa.

For the production of C12O, 5 L medium was inoculated with 10% culture inoculum and 600 ppm phenol was added as an inducer. The crude extract incubated with catechol did not show any absorbance at OD375 nm (detection of 2-hydroxymuconic semialdehyde) in contrast to an increased absorbance at OD260 nm (detection of cis-cis-muconic acid). The results concluded that the crude cell extract exhibited catechol 1,2-dioxygenase activity. Using the extinction coefficient of 14,800 µM/min/cm for muconic acid described above, the OD at 260 nm was converted to µM of product released. Thus, the specific activity of C12O from the crude extract was found to be 2.23 U/mg of protein. The crude cell extract concentrated and loaded in an ANX anion exchange purification column showed peaks at OD = 280 nm (Supplementary Material Figure S1). The fractions showing enzyme activity were pooled together, concentrated using a spin column, and again loaded on a gel filtration chromatography column packed with a Sephacryl HR100 matrix. Fractions A1 and A2 were collected and assayed for C12O activity (Supplementary Material Figure S2). Fractions A1 and A2 showed the presence of single-band protein on SDS-PAGE (Figure 1). The protein purified to 1.5-fold showed a specific activity of 2.02 U/mg of protein (Table 1).

### 2.2. Optimum pH and pH Stability of Purified C12O

**Figure 2:**
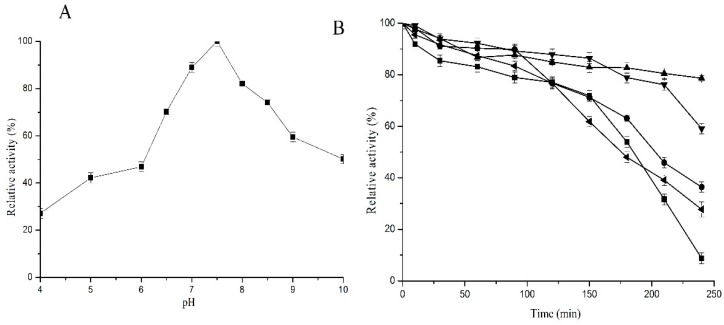
Optimum pH and pH stability of C12O from *Pc*UFB2. (**A**) 0.13 µg of enzyme incubated at different pH and assayed for enzyme activity; (**B**) pH stability profile of C12O at pH 4 (■), pH 6 (●), pH 7(▼), pH 8 (▲), and pH 10 (◄).

Purified C12O showed optimum activity at pH 7.5 (Figure 2A), retaining 84% and 80% of its activity at pH 7 and 8, respectively. Incubation of the enzyme for a period of 240 min and assayed at its optimum conditions showed that the enzyme was stable until 240 min at pH 8, retaining 79% activity. At pH 4, the enzyme lost about 50% activity after 180 min and 90% activity after 240 min. At pH 6, the enzyme retained 63% of its activity after 180 min and lost about 64% activity in 240 min. The enzyme was quite stable at pH 7, retaining 86% activity after 150 min and about 60% after 240 min (Figure 2B).

### 2.3. Optimum Temperature and Temperature Stability

**Figure 3:**
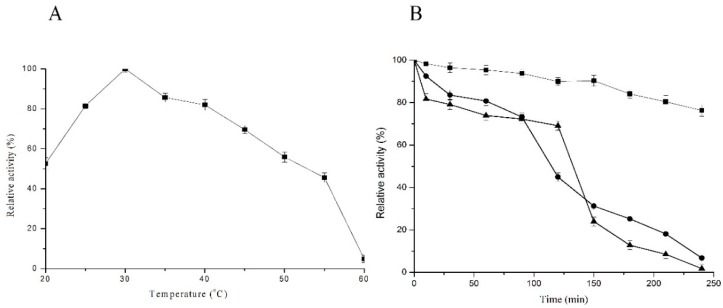
Optimum temperature and temperature stability of C12O from *Pc*UFB2. (**A**) 0.13 µg of enzyme incubated at a different temperatures and the activity was measured at optimum pH (7.5); (**B**) The temperature stability of C12O at 30 °C (■), 50 °C (●), and 70 °C (▲).

Purified C12O showed an optimum activity at 30 °C (Figure 3A), with 81% and 82% of its activity retained at 25 °C and 35 °C, respectively. The enzyme was stable until 120 min at 30 °C and lost only 5% activity even after incubation at 240 min. C12O was found to be unstable at 70 °C with 60% activity loss within 125 min and about 90% activity loss after 240 min. At 50 °C there was 50% loss of activity after 100 min, and the activity drastically decreased after 240 min with only 10% of activity remaining (Figure 3B).

### 2.4. The Kinetic Properties of C12O

The Lineweaver–Burk plots fitted in the Michaelis–Menten equation showed the *K*_m_ and *v*_max_ values of 35.76 µM and 16.67 µM/min, respectively (Supplementary Material Figure S3). The enzyme (0.13 µg) became saturated with 200 µM of catechol, showing the maximum reaction velocity constant until 500 µM of catechol. The curve did not indicate any type of substrate inhibition of the enzyme.

### 2.5. Effects of Metals and Inhibitors on C12O Activity

The effects of metals and inhibitors on purified C12O activity were evaluated by incubating 100 microliters (0.13 µg) of enzyme with a specific concentration of metals and inhibitors. In the presence of β-mercaptoethanol and EDTA, the enzyme showed 59% and 58% residual activity, respectively. C12O activity was drastically inhibited in the presence of metal ions like Cu^2+^, and Hg^2+^ with residual activity of 33% and 15%, respectively. The surfactants: Tween 20 and Tween 80 reduced the activity by only 20% and 4%, respectively. The protein denaturing agent SDS reduced activity to 10%, almost deactivating the enzyme (Table 2). 

### 2.6. Substrate Specificity of C12O

C12O showed more affinity to catechol as compared to other substrates (Table 3). The enzyme could catalyze phenol efficiently, showing 72% of residual activity as compared to catechol. C12O showed only 25%, 21%, and 51% of residual activity in the presence of 4-nitrophenol, 1,2,4-benzenetriol, and 2,4-DCP, respectively. C12O did not show any activity in the presence of 3-methylcatechol and 4-methylcatechol. The specific homogentisate 1,2-dioxygenase (H12D) activity was found to be 60 nmol^−1^·min^−1^·mg^−1^. The H12D activity could not be compared with other substrates as the products measured are different. 

### 2.7. Amplification and Detection of *C12O* in *Pc*UFB2

To confirm the presence of the *C12O* gene in *Pc*UFB2, primers were designed, and PCR experiments were performed. The results showed the amplification of the expected amplicon size of 467 bp as visualized on 1% agarose gel (Supplementary Material Figure S4).

### 2.8. ES-MS and Amino Acid Sequence Determination

The pure protein band from SDS-PAGE digested with trypsin generated six spectra matched with the gentisate oxidizing enzyme H12D (accession number: A0A0G3GN46, UniProt). H12D is involved in the catabolism of homogentisate (2,5-dihydroxyphenylacetate), a central intermediate in the degradation of phenylalanine and tyrosine. It catalyzes the oxidative ring cleavage of the aromatic ring of homogentisate to yield maleylacetoacetate. *Pseudomonas chlororaphis* accession number A0A0G3GN46 searched on www.Uniprot.org led to the depiction of an amino acid sequence of H12D. The protein featured an RmlC-like cupin domain superfamily (IPR011051). RmlC is a dimer, with each monomer formed from two beta-sheets arranged in a beta-sandwich, where the substrate-binding site is located between the two sheets of both monomers (http://www.ebi.ac.uk/interpro/entry/IPR011051). 

### 2.9. Template-Based Structure of Homogentisate 1,2-Dioxygenase

**Figure 4:**
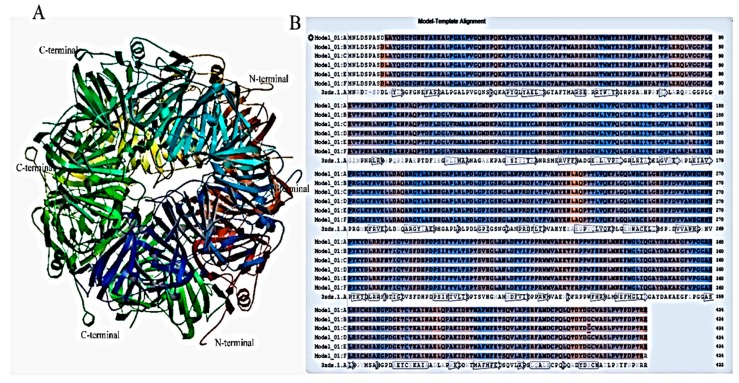
The model constructed by homology modeling at a SWISS-MODEL workspace using homogentisate 1,2-dioxygenase (H12D) from *Pseudomonas chlororaphis* as the template. (**A**) The predicted tertiary structure of H12D from *Pc*UFB2 deduced from 3zds.1.A; (**B**) Homology alignment of probable H12D from *Pc*UFB2 (Model_1) with H12D from *Pseudomonas chlororaphis* (3zds.1.A).

The amino acid sequence of H12D was retrieved from UniProt (A0A0G3GN46) and submitted at SWISS-MODEL. The predicted Model 3zds.1.A showed 81.06% homology with H12D from *Pseudomonas chlororaphis* (Figure 4A,B). The active site of H12D comprises the residues His292, His335, His365, His371, and Glu341. The active sites residues Glu341, His335, and His371 bind to homogentisate via the Fe^2+^ atom. His292 binds the hydroxyl group of the aromatic ring, and His365 binds to Glu341 via hydrogen bonding for amino acid stability [19].

### 2.10. Predicted Biophysical Properties of C12O

The biophysical parameters of the enzyme were determined by using the ExPaSy server. The parameters on the ExPaSy server included the number of amino acids, molecular weight, theoretical pI (isoelectric point, the pH at which a protein has no net charge), extinction coefficients (M^−1^ cm^−1^), estimated half-life, instability index (II), aliphatic index (AI) and grand average of hydropathicity (GRAVY). There were 314 amino acids present in C12O from *Pc*UFB2, with predicted molecular weight of 34.58 kDa and pI of 5.18, showing that C12O is acidic in nature. The extinction coefficient of the enzyme was found to be 30,035 M^−1^cm^−1^. The estimated half-life of C12O in *E. coli* in vitro was calculated to be greater than 10 h, showing its stability in prokaryotic cells. The instability index of the enzyme (39.92) showed that C12O was stable in vitro. The aliphatic index above 70 indicated that C12O is thermostable. C12O showed negative GRAVY (−0.477) indicating that the enzyme is hydrophilic.

## 3. Discussion

In this study, catechol 1,2-dioxygenase (C12O) from *Pseudomonas chlororaphis* strain UFB2 (*Pc*UFB2) was purified to 1.5-fold with a 13.02% yield. C12O showed a molecular weight of 35 kDa. PCR experiments also confirmed the presence of the *C12O* gene in *Pc*UFB2. Other studies have shown that C12Os from *Pseudomonas* spp. include homodimers and homo-tetramers and fall in the molecular weight range 20–40 kDa [15,20]. In this study, the optimum pH and temperature of C12O were found to be pH 7.5 and 30 °C, respectively. C12O from *Rhodococcus* sp. NCIM 2891 also showed optimum activity at pH 7.5 and 30 °C [21]. C12Os from other microorganisms have been shown to be optimum at different conditions. For example, the optimum activity for C12O was recorded at pH 8 and 37 °C for *Acinetobacter* sp. Y64 strain [22]; pH 7 and 30 °C for *Gordonia polyisoprenivorans* [11]; and pH 7.5 and 35 °C for *Pseudomonas putida* strain N6 [23]. However, an optimum temperature of 40 °C has been reported for C12O in *Pseudomonas aeruginosa* KB2 and *Candida albicans* TL3 strains [14,24]. C12O from *Mycobacterium fortuitum* immobilized on different surfaces have been reported to have an elevated optimum temperature of 45 °C.

The Lineweaver–Burk plot showed the *K*_m_ and *v*_max_ values of 35.76 µM and 16.67 µM/min, respectively. The *v*_max_ for C12O was about 105-fold higher than the previously reported *v*_max_ of 1218.8 U/mg for C12O from *Stenotrophomonas maltophilia* KB2 [25]. The *v*_max_ for C12O from *Rhodococcus opacus* 1CP and *Rhodococcus opacus* 6a were found to be 9.6 µM/min and 55.5 µM/min, respectively [26]. The *K*_m_ value in this study was higher than previously reported [21,24,25], indicating that the enzyme has less affinity for catechol. 

Investigation of the effect of metal ions and detergent on C12O revealed that the enzyme activity was significantly inhibited by heavy metals like Cu^2+^ and Hg^2+^ and the denaturing agent SDS. However, EDTA, Tween 20, Tween 80, and β-mercaptoethanol also showed 42%, 20%, 4%, and 41% inhibitory activity, respectively. Similar results were reported for a C12O from *Rhodococcus* sp. NCIM 2891, which was also inhibited by Cu^2+^ and Hg^2+^ [21]. However, a different trend in the effect of inhibitors and metals on C12O from *Candida albicans* TL3 was reported, where CuSO_4_ was found to not affect the activity of the enzyme [14], contrary to the observation in the current study. 

C12O elucidated a wide range of substrate specificity, showing high affinity for catechol and homogentisate. C12O showed low affinity for 2,4-dichlorophenol, 1,2,4-benzenetriol, 4-nitrocatechol, and phenol relative to catechol. Studies have reported that C12Os from different microorganisms have different substrate specificity. C12O from *Stenotrophomonas maltophilia* KB2 catalyzed 3-methylcatechol and 4-methylcatechol with 50% less efficiency as compared to catechol, while 2,4-dichlorophenol showed 74% relative activity [25]. C12O from *Pc*UFB2 was induced more efficiently when catechol was used as a substrate. Similar findings were reported where C12O from *Acinetobacter* sp. DS002 was induced by catechol, 1,2,4-benzenetriol, and 4-nitrocatechol, while 3-methylcatechol and 4-methylcatechol could not induce the expression of the enzyme [27]. In another study, 3-methylcatechol resulted in the induction of very low activity in *Acinetobacter* sp. Y64 (2% as compared to catechol), 1,2,4-benzenetriol, and 4-nitrocatechol, but 4-methylcatechol was able to induce high enzyme activity (80% as compared to catechol) [22]. In *Aspergillus awamori*, 2,4-dichlorophenol could not induce C12O [28]. Studies have shown that C12O expressed in *Rhodococcus*, *Ralstonia*, and *Pseudomonas arvilla* has a broad substrate specificity [27,29]. 

The purified protein band digested with trypsin and followed by ES-MS analysis resulted in the depiction of the amino acid sequence. The results showed that C12O activity may be due to homogentisate 1,2-dioxygenase (H12D) expressed in *Pc*UFB2. The amino acid sequence of H12D showed 81.06% homology with model 3zds.1.A on SWISSPROT, which could lead to its structure prediction. It was also confirmed that H12D is an intradiol enzyme and catalyzes homogentisate to maleylacetoacetate. It has been reported that the ring cleavage in homogentisate is a multiple-step process. The initial step is the coordination of carbonyl and ortho phenol oxygens by Fe^2+^ to His335, His371, and Glu341 [25]. The structure shows an octahedral coordination for Fe^2+^ with two histidine residues (His331 and His367), a bidentate carboxylate ligand (Glu337), and two water molecules. Homogentisate binds as a monodentate ligand to Fe^2+^, and its interaction with Tyr346 results in the folding of a loop over the active site, effectively shielding it from solvent [30]. In *Pseudomonas putida*, homogentisate cleavage is facilitated by H12D, producing maleylacetoacetate. The maleylacetoacetate is isomerized to fumarylacetoacetate by maleylacetoacetate isomerase. Fumarate and acetoacetate are then formed from the catalysis of fumarylacetoacetate by fumarylacetoacetate hydrolase [31]. Oxygen atom (O_2_) binds to the iron atom and reacts with the aromatic ring [25,31]. Most intradiol dioxygenases enzymes have an N-terminal domain with five α-helices and a C-terminal domain consisting of β-sheets. A similar structure of C12O from *Pseudomonas putida* N6 has been reported [23]. It is probable that in *Pc*UFB2, catechol oxidation is facilitated by H12D, leading to the production of maleylacetoacetate. To the best of our knowledge, this is the first report of H12D oxidizing catechol and other related substrates (Table 3).

The biophysical properties reported in this study were found to be like those previously reported for C12O from *Pseudomonas* spp., where molecular weight ranged from 22 to 40 kDa and pI ranged from 4 to 8 [15]. The instability index differed and ranged from to 35 to 47, which indicates that the enzyme can be stable or unstable depending on its type and location. The aliphatic index ranging from 70 to 85 showed that the enzyme can be thermostable and hydrophilic, showing a negative GRAVY value. The bioinformatics and biophysical study of C12O in other *Pseudomonas* spp. showed that the number of amino acids, molecular weight, and pI had the ranges 314–327, 34–36, and 4–11 kDa, respectively. Almost all the C12O reported in *Pseudomonas* spp. are found to be acidic in nature, except from *Pseudomonas chlororaphis* strain PCL1606 showing pI 11.37, and basic in nature. The estimated half-life for all C12O (*E. coli* in vitro) was greater than 10 h. The aliphatic index of the enzyme ranged from 52 to 79, with the lowest value of 52.57 obtained for *Pseudomonas chlororaphis* strain PCL1606. 

## 4. Materials and Methods

### 4.1. Sample Collection, Enrichment, and Isolation of Bacterial Isolates

Sample collection, media preparation, enrichment and isolation of cultures were performed as described previously with some modifications [32]. An activated sludge sample with known history of contamination with chlorinated organic compounds was collected from the New Germany wastewater treatment plant located in Durban, South Africa. Samples were collected in 500 mL bottles and immediately stored at 4 °C until used for the culture enrichment set-up. The mineral salt medium (MSM) used for the culture enrichment comprised (in mg/L): KH_2_PO4, 800; Na_2_HPO_4_, 800; MgSO_4_·7H_2_O, 200; NH_4_SO_2_, 500. The pH was adjusted to 7.5 using 2 M NaOH prior to autoclaving at 121 °C for 15 min. One mL of trace metal which comprised (in mg/L): FeSO_4_·7H_2_O, 5; ZnSO_4_·7H_2_O, 4; MnSO_4_·4H_2_O, 0.2; NiCl·6H_2_O, 0.1; H_3_BO_3_, 0.15; CoCl_2_·6H_2_O, 0.5; ZnCl_2_, 0.25; EDTA, 2.5 was added by syringe filter (0.2 µm pore) into 1 L MSM. Ten percent of the activated sludge sample was inoculated into MSM that was supplemented with 40 ppm of 2,4-dicholorophenol (2,4-DCP) in a 250 mL Erlenmeyer flask and incubated at 30 °C and shaken at 150 rpm for a week. Sub-culturing in fresh MSM was carried out until a stable and consistent culture was obtained. Aliquots from each culture were spread on MSM agar plates supplemented with 40 ppm of 2,4-DCP and incubated at 30 °C until visible growth of the microorganisms was observed. Pure cultures were obtained by streaking individual morphologically different colonies on nutrient agar plates. The pure colonies were stored at −70 °C as 20% (*v*/*v*) glycerol stocks. 

### 4.2. Identification and Phylogenetic Analysis of the Bacterial Isolate

The 16S rRNA gene was amplified from the purified genomic DNA (Genomic DNA Purification Kit, Thermo Scientific, Waltham, MA, USA) of the bacterial isolate as a template using the universal primer pair: 63F-5′-CAGGCCTAACACATGCAAGTC-3′ and 1387R-5′-GGGCGGTGTGTACAAGGC-3′ [33]. Ten microliters of PCR reaction mixture contained: 1 µL buffer (10×), 0.6 µL MgCl_2_ of (25 mM), 0.2 µL of 200 µM dNTPs, 0.2 µL of each primer (10 µm), 0.05 µL of AmpliTaq polymerase, ∼20 ng DNA template, and 7.3 µL autoclaved double distilled water. The PCR conditions were as follows: 95 °C for 5 min, (1 cycle) 95 °C for 30 s, 55 °C for 1 min, and 72 °C for 1 min (35 cycles), and a final elongation at 72 °C for 10 min (1 cycle). The amplified 16S rRNA gene was sequenced at Inqaba Biotechnical Industries (Pretoria, South Africa) and submitted to the National Centre for Biotechnology Information (NCBI) database (http://www.ncbi.nlm.nih.gov/blast/ using the blastn algorithm) for the identification of organisms. The 16S rRNA gene sequences were retrieved from NCBI and the phylogenetic tree was constructed by a rooted neighbor-joining method using DNAMAN (Lynnon Corporation, CA, USA; v.7 Demo version). The numbers on branching points are bootstrap values with 1000 replicates (values < 95% were not included) (Supplementary Material Figure S5).

### 4.3. Preparation of Crude Extracts for Catechol 1,2-Dioxygenase (C12O) and Catechol 2,3-Dioxygenase (C23O) Activity

The crude extract was prepared by growing the bacterial cells for 36 h in mineral salt medium (MSM) comprising (g/L): K_2_HPO_4_, 2.75; KH_2_PO_4_, 0.1; NH_4_Cl, 0.2; MgSO_4_·7H_2_O, 0.01; CaCl_2_·2H_2_O, 1.0; NH_4_Cl, 0.5, and yeast extract, 1.0. The pH was adjusted to 7.0 with 2 M NaOH prior to autoclaving at 121 °C for 15 min. One milliliter of trace metal solution composed of (mg/L): FeSO_4_·7H_2_O, 5; ZnSO_4_·7H_2_O, 4; MnSO_4_·4H_2_O, 0.2; NiCl·6H_2_O, 0.1; H_3_BO_3_, 0.15; CoCl_2_·6H_2_O, 0.5; ZnCl_2_, 0.25; EDTA, 2 was added by syringe filter (0.2 µL pore) into 1 L of the MSM. *Pc*UFB2 cells were grown in nutrient broth overnight at 30 °C and the culture was standardized to OD = 1 at 600 nm. Ten percent of the standardized culture was inoculated into the above-described MSM supplemented with 600 ppm of phenol as a sole carbon and energy source. The inoculated medium was incubated at 30 °C for 36 h, shaking at 150 rpm. The cells were harvested at the late exponential phase of growth by centrifugation at 10,000× *g* for 15 min at 4 °C. The cells were washed twice with 50 mM sodium phosphate buffer, pH 7.5 (containing 1 mM EDTA and 1 mM β-mercaptoethanol to halt the protease activity). A total of 24 g of the cell pellet was collected and re-suspended in 100 mL of the same buffer. Cell-free extracts were prepared by lysing the pellet by sonication with a 400 Ultrasonicator (OMNI International), 8 cycles each with a pulse of 30 s on/off for 4 min. The cell extract was centrifuged at 20,000× *g* for 30 min at 4 °C. The clear supernatant was kept on ice to prevent inactivation of the enzymes and used as a crude extract for enzyme assays, while the remaining extract was kept in −20 °C for further studies [4].

### 4.4. C12O and C23O Activity Assay

C12O and C23O activity were assayed in a 1 mL reaction mixture as described previously [4,32]. The reaction mixture contained 10 mM of catechol in 50 mM sodium phosphate buffer (pH 8.0). The reaction was initiated by adding 100 µL of crude enzyme into the reaction mixture and incubated for 30 min at 30 °C. Buffer plus the enzyme only, and buffer plus substrate without the enzyme were used as controls. The initial and final absorbance at 260 and 375 nm were measured using a UV–Vis spectrophotometer (UV-1800, Shimadzu, Kyoto, Japan) fitted with a CPS-240A temperature controller unit set at 30 °C. One unit of enzyme activity was defined as the amount of the enzyme that produced 1 µM of either *cis,cis-*muconic acid at 260 nm (catechol 1,2-dioxygenase) or 2-hydroxymuconic semialdehyde at 375 nm (catechol 2,3-dioxygenase) under standard assay conditions. Enzyme activity was calculated using the equation: enzyme activity (µm of product formed/min) = {(ε × *L*/*V*) (ΔOD/min)}, where ΔOD is the change in optical density at the different wavelengths; ε is the molar extinction coefficient of the product; “*V*” is the reaction volume, and “*L*” is the path length (mm). The molar extinction coefficients 16,800 mM^−1^·cm^−1^ (muconic acid) and 14,700 mM^−1^·cm^−1^ (2-hydroxysemialdehyde) were used to determine the activities for catechol 1,2-dioxygenase and catechol 2,3-dioxygenase, respectively [4,32].

### 4.5. Purification of C12O

The enzyme was purified by using anion exchange and gel filtration chromatography. For anion exchange, 1 mL anion exchange HiTrap ANX column was equilibrated with five column volumes (CVs) (1 CV = 5 mL) of 20 mM sodium sulfate buffer (pH 8) and 1 mL (200 µg total protein) of the sample was loaded into the column. The unbound proteins were washed with 5 CV of 20 mM sodium sulfate buffer (pH 8). The proteins bound to the matrix were eluted with 10 CV of a 0–1.0 M linear gradient of NaCl in 20 mM sodium sulfate buffer (pH 8). The eluted proteins were collected as 1 mL fractions using the AKTA purifier 100-P950 automated fraction collector at a flow rate of 1 mL/min. The fractions showing C12O activity were concentrated with chilled acetone (200 µL of fractions and 800 µL acetone) for 2 h at −70 °C, and the samples were loaded on 12% SDS-PAGE to confirm purity and homogeneity. The fractions showing the C12O activity were pulled together and concentrated using an Amicon Ultra-15 centrifugal filter unit (MW cut off 10 kDa). One milliliter of sample (0.36 mg of total protein) was again loaded in a 35 mL (1 CV) gel filtration column manually packed with Sephacryl HR 100 matrix (from Sigma-Aldrich, St Louis, MO, USA) and equilibrated with 2 CV of 20 mM sodium sulfate buffer (pH 8) and collected as 2 mL fractions using the AKTA purifier 100-P950 automated fraction collector at a flow rate of 0.5 mL/min. The fractions showing C12O activity were pulled together and concentrated using an Amicon Ultra-15 centrifugal filter unit (MW cut off 10 kDa). The fractions were concentrated with chilled acetone (200 µL of fractions and 800 µL acetone) for 2 h at −70 °C and the samples were loaded on 12% SDS-PAGE to confirm purity and homogeneity [34].

### 4.6. Determination of Optimum pH and Temperature

The optimum pH of purified C12O was determined by setting up a reaction in different buffers as follows: 50 mM citrate-phosphate buffer (pH 4–6.5), sodium phosphate buffer (pH 7.0–8.0), and Tris-phosphate buffer (pH 8.5–10) [6]. The optimum temperature was determined by incubating the reaction mixture for 30 min at 20, 25, 30, 35, 40, 45, 50, and 60 °C. The reaction was set up as described above.

### 4.7. Temperature and pH Stability of C12O

To determine the pH stability of C12O, an adequate volume of the enzyme was incubated in buffers: 50 mM citrate-phosphate buffer (pH 4 and 6), 50 mM sodium phosphate buffer (pH 7.0 and 8.0), and 50 mM Tris-phosphate buffer (pH 10) at a designated time (0–2 h). One hundred microliters (0.13 µg) of the enzyme aliquots were withdrawn at different time intervals and the enzyme reaction assay was set up as described above. The relative enzyme activity at different pHs was represented related to the initial activity. To determine the temperature stability, an adequate volume of the enzyme (0.13 µg) was incubated at 30, 50, and 70 °C in sodium phosphate buffer (pH 8.0) for 0–2 h. One hundred microliters (0.13 µg) of the enzyme aliquots were withdrawn at different time intervals and the reactions were set up as described above. The residual enzyme activity at different temperatures was represented relative to the initial activity.

### 4.8. Determination of the Enzyme Kinetic Parameters

The kinetic parameters were determined by measuring the initial rate of enzymatic activity. One hundred microliters (0.13 µg) of the enzyme was incubated with sodium phosphate buffer (pH 8) containing catechol (0–500 µM) at 30 °C for 30 min. The Lineweaver–Burk plot was constructed by plotting the reciprocal of the rate of substrate hydrolysis (1/V) against the reciprocals of the substrate concentrations (1/[S]). The *v*_max_ and *K*_m_ values were determined by fitting the data in Michaelis–Menten equation using ORIGIN 8 pro (Evaluation version). 

### 4.9. Effects of Metals and Inhibitors on C12O Activity

One hundred microliters (0.13 µg) of purified C12O was incubated separately with 0.1 mM of β-mercaptoethanol, EDTA, CuSO_4_, HgCl_2_, Tween 20, Tween 80, and sodium dodecyl sulfate (SDS), and the enzyme assay was performed as described above.

### 4.10. Substrate Specificity of C12O

To determine the substrate specificity of C12O, stock solutions of various substrates (i.e., phenol, 4-nitrocatechol, 3-methylcatechol, 4-methylcatechol, 1,2,4-benzenetriol, catechol, and 2,4-dichlorophenol) were prepared in 50 mM sodium phosphate buffer (pH 8), except homogentisate was prepared in 20 mM MES (pH8). One hundred microliters (0.13 µM) enzyme was added to the 0.1 mM of the substrates except for catechol (0.2 mM) to initiate the reaction, and the assay was performed as described above. Homogentisate 1,2-dioxygenase (H12D) activity was monitored by spectrophotometric method [35,36]. The assay contained 1 mL of buffer and 200 µM homogentisate, and the production of maleylacetoacetate was monitored at 330 nm {ε = 10.1 mM^−1^·cm^−1^, 20 mM MES, 80 mM NaCl (L = 0.1), pH 8, 25 °C}.

### 4.11. Determination of Amino Acid Sequences of the Purified C12O

The pure protein (50 µg) was loaded onto 12% SDS-PAGE and stained with Coomassie blue R250. The protein band was excised carefully and digested with trypsin and fragments were analyzed by electrospray mass spectrometry (at CAF, Stellenbosch University, Stellenbosch, South Africa). The raw files generated by the mass spectrometers were imported into Proteome Discoverer v1.3 (Thermo Scientific, USA) and SearchGUI v. 3.2.18 and processed using the Mascot 1.3. Algorithm (Matrix Science) as well as the Sequest algorithm to get the peptides generated from the enzyme. The database from tryptic digestion was analyzed by PeptideShaker (version 1.16.9) for homology of C12O. The FASTA sequence was then used for the functional features at UniProt (http://www.uniprot.org/). 

### 4.12. Prediction of Biophysical Properties and Three-Dimensional Structure

The biophysical properties of the protein were determined using ExPASy server, while structure prediction of the enzymes was carried out using SWISS-MODEL workspace (http://swissmodel.expasy.org). The default parameters used for performing the automated SWISS-MODEL were used as explained previously [34] and elaborated at (http://swissmodel.expasy.org/docs/help) webpage. The modeled PDB files were submitted to online tool RAMPAGE for Ramachandran plot analysis to check the quality and validation of the predicted models [34].

### 4.13. Amplification and Detection of C12O in Pc*UFB2*

To detect and amplify *C12O* gene in *Pc*UFB2, the whole gene sequence was retrieved from https://www.ebi.ac.uk/ena/data/view/AKK00187. The sequence was exploited to design forward 5′-ATGGCTAACATTCTCGGCGG-3′ and reverse primers 5′-TGGCCGAGTTTGTAACAACGG-3′ amplifying a 467 bp region. PCR and conditions were used as described above except for the annealing temperature at 62 °C.

## 5. Conclusions

This study covered the purification, characterization, and three-dimensional structure prediction of catechol 1,2-dioxygenase from *Pc*UFB2. The enzyme was found to be identical to the H12D which hydroxylates homogentisate to maleylacetoacetate. The kinetics parameters of the enzyme show that it has high affinity for catechol and homogentisate. Enzyme purification data, SDS-PAGE, and PCR experiments confirmed the presence of catechol oxidizing enzyme in *Pc*UFB2. To the best of our knowledge, this is the first report of an enzyme showing both C12O and H12D activity. The characteristics of the purified C12O showed that the enzyme may have application in the bioremediation of pollutants. 

## Figures and Tables

**Table 1:** Purification of catechol 1,2-dioxygenase (C12O) from *Pseudomonas chlororaphis* strain UFB2 (*Pc*UFB2).

Step	Total Activity (U/mL)	Total Protein (mg/mL)	Specific Activity (U/mg)	Yield (%)	Purification Fold
Crude	10.34	4.64	2.23	100	1
Anion Exchange Chromatography	5.16	1.75	2.91	57.7	1.3
Gel Filtration Chromatography	2.02	0.59	3.42	13.02	1.5

**Table 2:** Effects of metals and inhibitors (1 mM) on the purified C12O from *Pc*UFB2.

Metal/Inhibitor/Detergent	Residual Activity (%) *
None (Control)	100.00 ± 0.04
β-Mercaptoethanol	59.00 ± 0.03
EDTA	58.00 ± 0.02
CuSO_4_	33.00 ± 0.01
HgCl_2_	15.00 ± 0.02
Tween 20	80.00 ± 0.02
Tween 80	96.00 ± 0.02
SDS	10.00 ± 0.01

* Residual activity of C12O represented the percentage of activity (U/mL) in the presence of various substrates (metals and inhibitors) as compared to the activity measured in the presence of catechol.

**Table 3:** Substrate specificity of purified C12O from *Pc*UFB2.

Substrates	Residual Activity (%) *
Catechol	100.00 ± 0.01
4-Nitrocatechol	25.00 ± 0.00
4-Methylcatechol	0.00 ± 0.00
3-Methylcatechol	0.00 ± 0.00
1,2,4-Benzenetriol	21.00 ± 0.02
Phenol	72.00 ± 0.02
2,4-Dichlorophenol	51.00 ± 0.00
Homogentisate	60 nmol^−1^·min^−1^·mg^−1 #^

* Residual activity of C12O represented as the percentage of U/mL activity in the in the presence of various substrates as compared to the activity measured in the presence of catechol. ^#^ cannot be calculated relatively as the products measured are different.

## Supplementary Materials

Supplementary materials can be found at http://www.mdpi.com/1422-0067/20/1/61/s1.

## Abbreviations

*Ps*UFB2	*Pseudomonas chlororaphis* strain UFB2
C12O	Catechol 1,2-Dioxygenase
C23O	Catechol 2,3-Dioxygenase
H12D	Homogentisate 1,2-Dioxygenase
