# Supplementary material for: Catechol 1,2-Dioxygenase is an Analogue of Homogentisate 1,2-Dioxygenase in Pseudomonas chlororaphis Strain UFB2
Source: Int J Mol Sci. 2018 Dec 24;20(1):61. doi: 10.3390/ijms20010061 (PMC6337169; doi:10.3390/ijms20010061)
Supplement: Supplementary file 1 [file ijms-20-00061-s001.pdf]

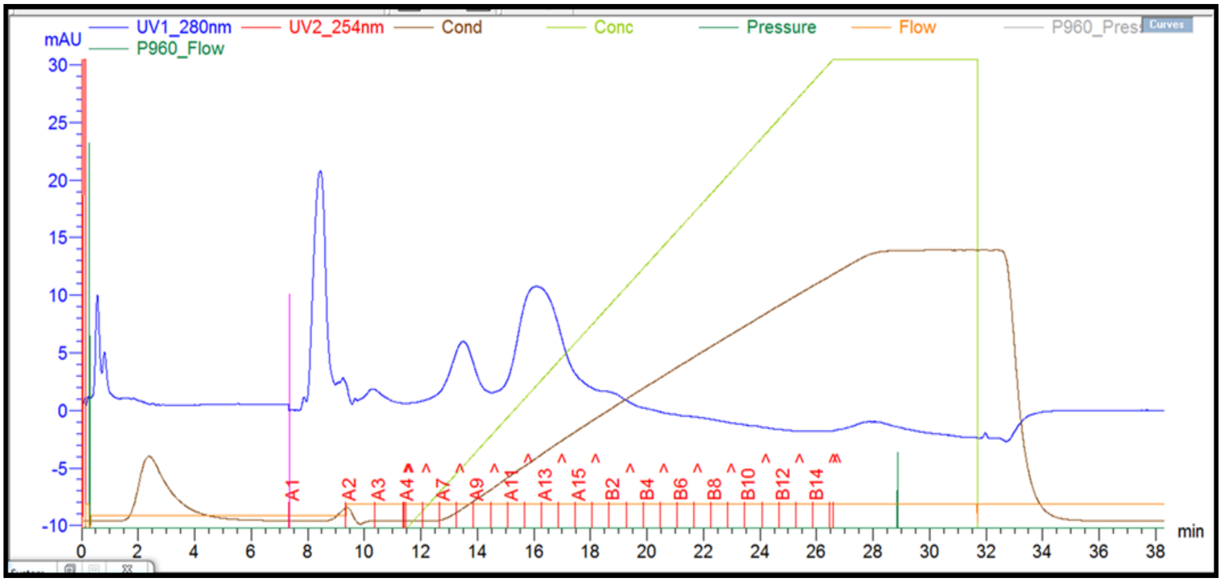


**Supplementary Material Figure S1:** Anion Exchange Chromatogram of crude extract loaded on ANX anion exchange column connected to AKTA100 purification system. Labels from A1-A14 shown the number of fractions collected.

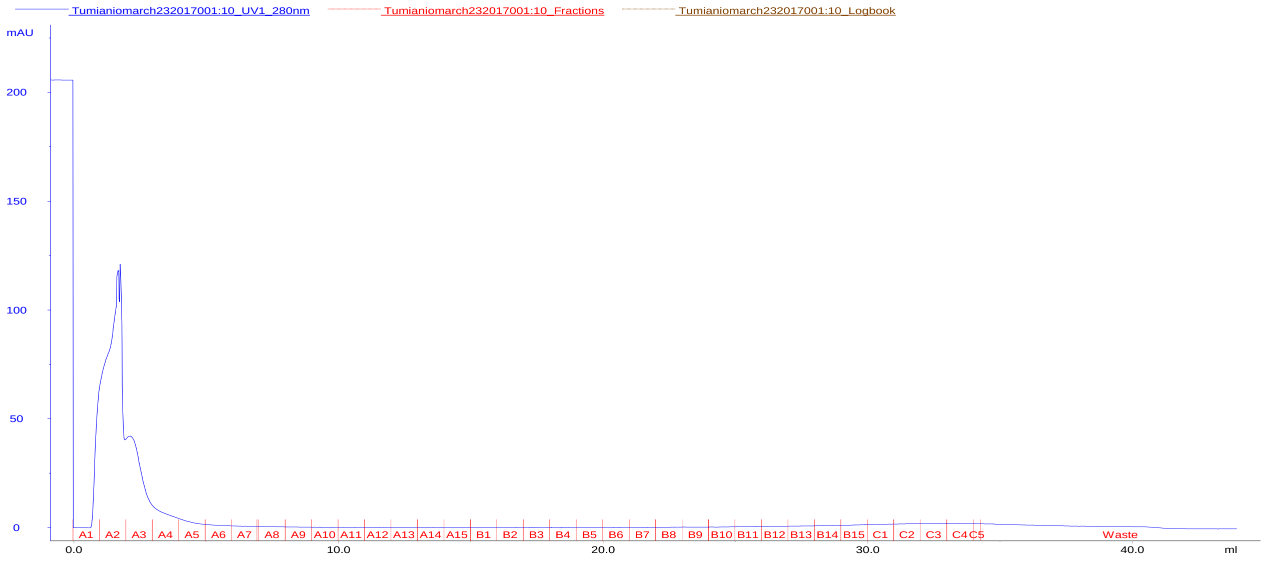


**Supplementary Material Figure S2:** Gel Filtration Chromatogram: Fraction collected after ANX anion exchange chromatography, pooled, concentrated and loaded on a column packed with Sepharyl 100 matrix. Labels from A1-C5 shows a number of the fraction collected.

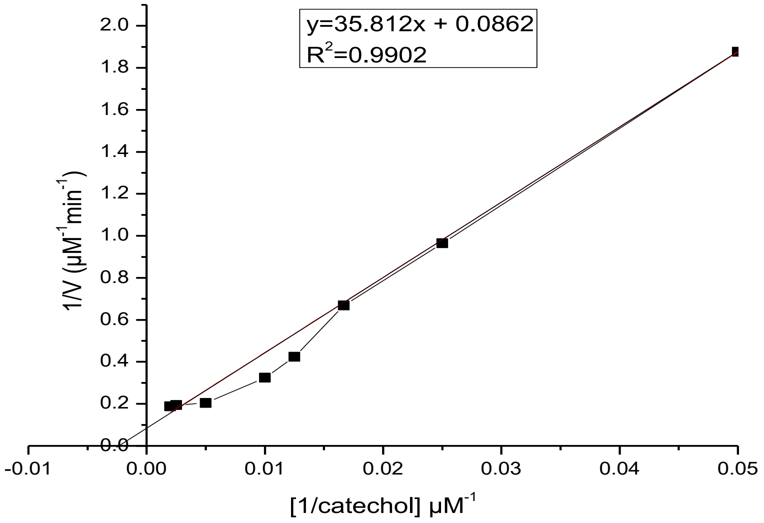


**Supplementary Material Figure S3:** Lineweaver-Burk double reciprocal plot of substrate saturation curve of catechol 1,2-dioxygenase. The double reciprocal plot was fitted to Michaelis-Menten equation to determine the values of $v_{max}$ and $K_m$.

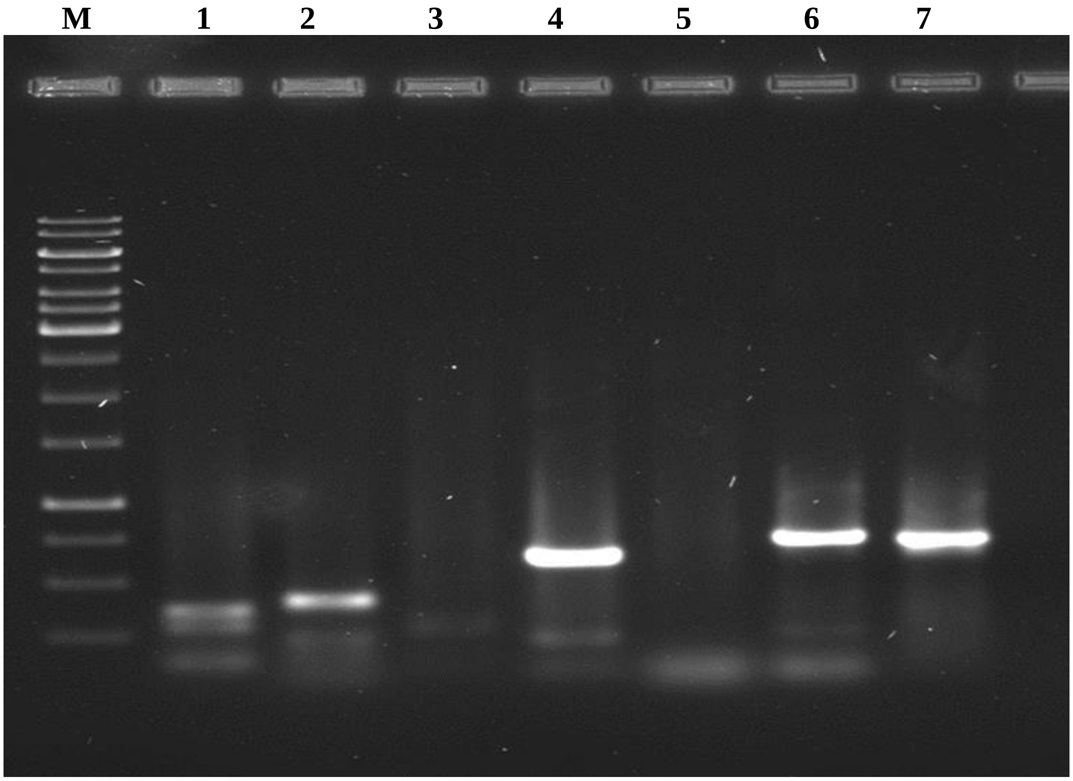


**Supplementary Material Figure S4:** Amplification of genes involved in the biodegradation of 2,4-DCP in *Pseudomonas chlororaphis* UFB2: M=1Kb DNA Marker, Lane 2= catechol 1,2-dioxygenase gene (467 bp).

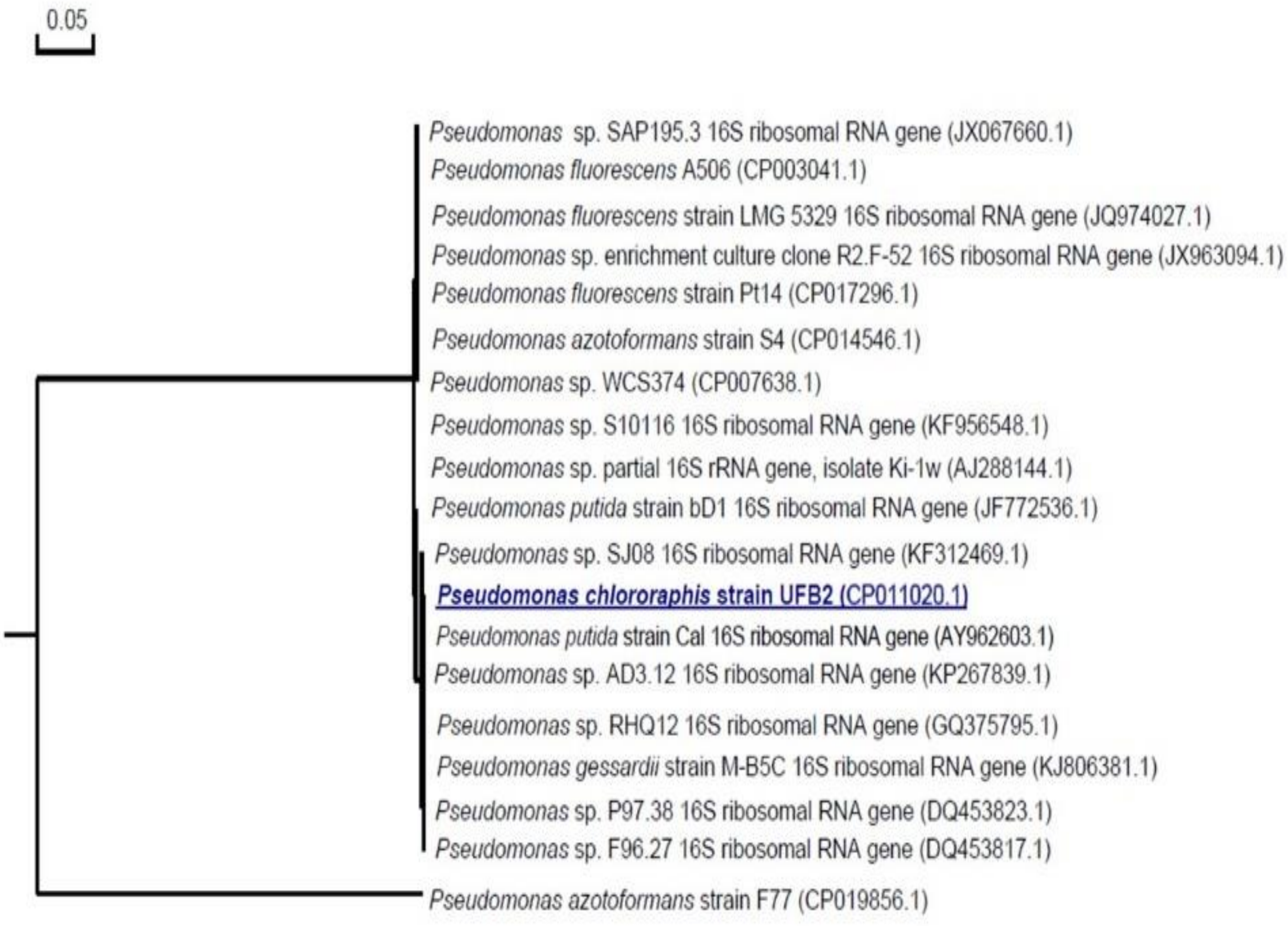


**Supplementary Material Figure S5**: Phylogenetic tree showing relatedness of *Pseudomonas chlororaphis* UFB2 isolated in this study to other reported 2,4-DCP and other phenolic compounds degrading *Pseudomonas* spp. The 16S rRNA sequences were retrieved from NCBI and the phylogenetic tree was constructed by rooted neighbour-joining method using DNAMAN (version 7), Lynnon Corporation, CA, USA (Demo version). The numbers on branching points are bootstrap values with 1000 replicates (values <95% were not included).
